# Incidental Renal Botryomycosis in a Nonfunctioning Kidney

**DOI:** 10.1155/2012/239093

**Published:** 2012-12-19

**Authors:** Mary Mathew, Bhavna Nayal, Bhawna Nagel, Joseph Thomas

**Affiliations:** ^1^Department of Pathology, Kasturba Medical College, Manipal University, Manipal 576104, India; ^2^Department of Urology, KMC, Manipal University, Manipal 576104, India

## Abstract

Botryomycosis is a unique form of bacterial infection, closely mimicking actinomycosis. The usual site of occurrence is the skin and renal botryomycosis is very rare. The most common organism is *Staphylococcus aureus* which can be identified using Gram stain and confirmed by culture. Early and accurate diagnosis can ensure appropriate antibiotic therapy. We present a young lady diagnosed to have incidental botryomycosis of the kidney, following nephrectomy.

## 1. Introduction

Botryomycosis is a rare chronic suppurative bacterial condition characterized by the presence of masses of fungus-like sulphur granules. This infection may involve the skin or visceral organs [1, 2]. To the best of our knowledge, very few cases of renal botryomycosis have been reported in the literature [3–5].

## 2. Case Report

**Figure 1:**
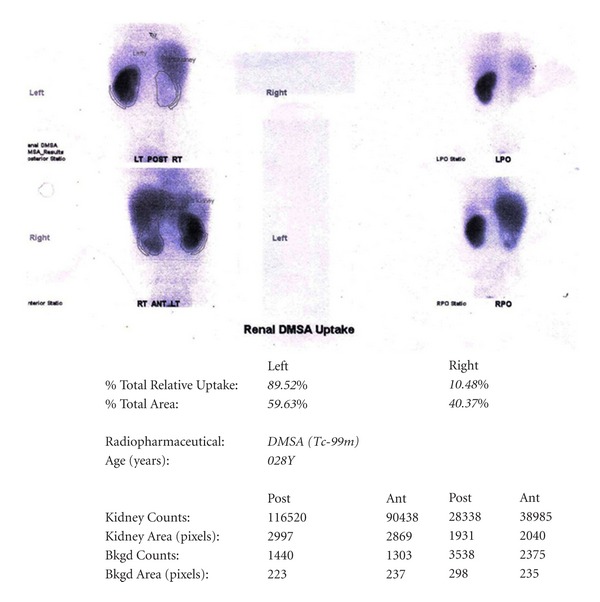
DMSA scan showing a poorly functioning right kidney.

**Figure 2:**
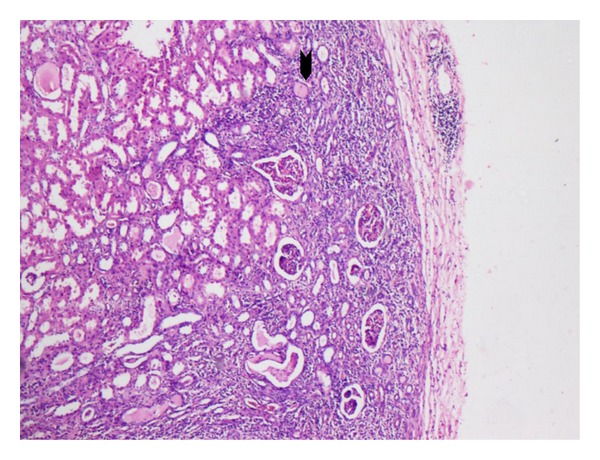
Microscopic section of the kidney showing features of chronic pyelonephritis with dense interstitial inflammatory infiltrate and atrophic tubules (arrow head). (H&E, ×10).

**Figure 3:**
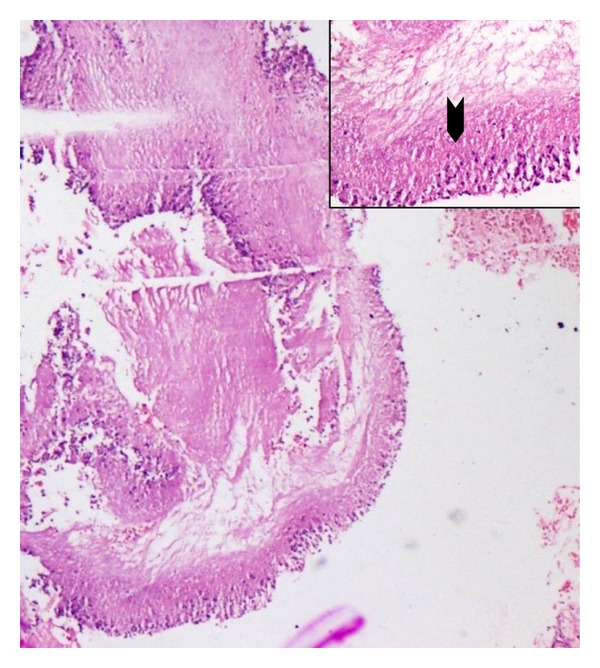
Splendore Hoeppeli phenomenon surrounding bacterial colonies with inflammatory cells (H&E, ×20) Inset, Splendore Hoeppeli phenomenon (arrow head) (H&E, ×40).

**Figure 4:**
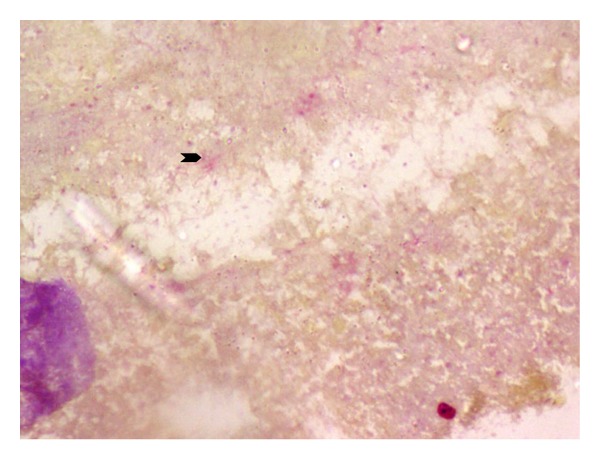
Gram negative bacilli in the abscess (arrow head) (Gram stain, ×40).

A 29-year-old nondiabetic lady presented with right loin pain and fever of two months duration. The urine microscopy revealed numerous red blood cells and bacteria, and serum urea and creatinine levels were normal. An intravenous pyelogram showed a staghorn calculus. A dimercaptosuccinic acid (DMSA) scan performed at the same visit showed a right hydronephrotic, poorly functioning kidney (Figure 1). A right percutaneous nephrostomy with double J stenting was performed and pus was obtained during the procedure. She was discharged with a right DJ stent left in situ. Six weeks later, she presented with persistent loin pain and diethylene triamine pentaacetic acid (DTPA) renogram showed a poorly functioning right kidney (19.29%). A right nephrectomy was undertaken and the specimen was sent for histopathological examination. Grossly, the right kidney appeared small and shrunken with cortical scarring and adherent capsule. Cut section of the kidney revealed cortical atrophy with a dilated pelvicalyceal system containing luminal necrotic debris. On histopathological examination, the renal interstitium showed fibrosis and dense inflammation composed of lymphocytes, plasma cells, neutrophils, eosinophils, and occasional multinucleated giant cells. The tubules were focally dilated and contained necrotic debris (Figure 2). Occasional glomeruli showed periglomerular fibrosis. The renal parenchyma showed abscesses containing Gram negative organisms surrounded by amorphous eosinophilic material suggestive of Splendore Hoeppeli phenomenon (Figures 3 and 4). These bacterial colonies were negative for Grocott-Gomori silver and Ziehl-Neelson stains. A final diagnosis of renal botryomycosis superimposed on chronic pyelonephritis was rendered. The patient was treated with cefuroxime (2nd generation cephalosporin) for two weeks and prulifloxacin (new generation fluroquinolone) for 3 days. On followup, two years hence, the patient is asymptomatic and her urine microscopy is normal.

## 3. Discussion

Botryomycosis is an uncommon chronic granulomatous bacterial infection, primarily affecting the cutaneous and subcutaneous tissue. Visceral involvement is unusual [2, 3]. The lung is the most common viscera involved; however botryomycosis of the liver, trachea, heart, bowel, and brain have also been documented [6]. Rare cases of renal botryomycosis have been reported in the literature (Table 1).

The most common causative agent of botryomycosis is *Staphylococcus aureus*, followed by *Pseudomonas aeruginosa*. Other bacterial etiological agents include *Micrococcus pyogenes*, *Streptococcus*, *Escherichia coli*, and *Neisseria* [2].

The factors implicated in the pathogenesis of botryomyocosis are impaired cellular immunity, virulence of the causative agents, phagocytic defects, and delayed hypersensitivity reactions [3]. Predisposing conditions include skin trauma, immunosupression due to diabetes mellitus, steroid therapy, or human immunodeficiency virus infection and cystic fibrosis [2, 3]. These factors were absent in this case.

The most important differential diagnosis of botryomycosis is actinomycosis. Both these lesions demonstrate Splendore-Hoepple phenomenon characterized by deposition of periodic acid-Schiff positive amorphous eosinophilic material around bacterial colonies [6]. Gram and Grocott-Gomori silver stain help is distinguishing these two entities. Gram stain identifies the causative bacterial agent in botryomycosis, which is confirmed by culture. These bacterial colonies are usually negative for Grocott-Gomori silver stain [6–8]. The present case revealed Gram negative bacilli. Specific subtyping could not be done due to lack of urine culture.

Furthermore renal botryomycosis can rarely present as a renal mass mimicking a neoplastic process and may pose a diagnostic dilemma. The diagnosis of botryomycosis if considered preoperatively on needle biopsy can be treated with appropriate antibiotic regimen, preventing the need for radical nephrectomy [1, 5]. In the present case, the indication for nephrectomy was a poorly functioning hydronephrotic kidney in which botryomycosis was detected incidentally. As the urine culture was not done, we cannot categorically state that the botryomycotic infection presented prior to percutaneous nephrostomy.

In conclusion, renal botryomycosis is a rare treatable condition and if diagnosed early and accurately can prevent unnecessary sacrifice of the kidney.

## Figures and Tables

**Table 1:** Previous reported cases of renal botryomycosis.

Author	Year	Age (years)	Sex	Clinical diagnosis	Organism isolated
Richmond and Mene [4]	1992	60	Female	Renal carcinoma	Gram-negative bacilli
François et al. [3]	1994	60	Female	Chronic bacterial infection	*E. coli *
Yörükoğlu et al. [1]	1998	57	Male	Renal carcinoma	*E. coli *
Sahin et al. [5]	2004	—	—	Renal mass	—
Present case	2012	29	Female	Non functioning kidney	Gram-negative bacilli

## References

[1] Yörükoğlu K, Ozer E, Sade M, Biberoğlu K, Kirkali Z (1998). Renal botryomycosis mimicking renal cell carcinoma. *Journal of Urology*.

[2] Makama JZ, Khan N, Makhanya NZ, Motswaledi H (2006). Botryomycosis. *South African Journal of Radiology*.

[3] François A, Bacri JL, Métayer J, Hemet J (1994). Renal botryomycosis. Clinicopathologic study of a case. *Annales de Pathologie*.

[4] Richmond I, Mene A (1992). Renal botryomycosis. *Histopathology*.

[5] Sahin MO, Erdem AC, Mungan MU, Kirkali Z, Sade M (2004). Benign lesions underwent radical nephrectomy for renal cancer. *Türk Üroloji Dergisi*.

[6] Gupta K, Das A, Radotra BD, Bhalla A (2008). Cardiac botryomycosis: an autopsy report. *Journal of Clinical Pathology*.

[7] Bersoff-Matcha SJ, Roper CC, Liapis H, Little JR (1998). Primary pulmonary botryomycosis: case report and review. *Clinical Infectious Diseases*.

[8] Tomb RR, Stephan F, Haddad A, Choucair J (2009). Cutaneous granular bacteriosis, a rarely diagnosed infection of the head and the neck. *Clinical and Experimental Dermatology*.

